# Money and mental health: a scoping review of financial variables, data sources, and analytical methods

**DOI:** 10.3389/fpubh.2026.1812845

**Published:** 2026-07-01

**Authors:** Oluwadara Adedeji, Andreas Balaskas, David Coyle, Keith Gaynor, Mark Matthews

**Affiliations:** 1 School of Computer Science, University College Dublin, Dublin, Ireland; 2 School of Psychology, University College Dublin, Dublin, Ireland

**Keywords:** finance, finhealth, machine learning, mental health, mental wellbeing, money, statistical analysis

## Abstract

**Background:**

The relationship between financial circumstances and mental health is well-established. New financial data sources such as bank transactions and digital payments offer new opportunities to better characterize this relationship. However, the prior financial data sources and analytical approaches have not been systematically reviewed. Understanding these is essential to guide future research and inform integrated interventions that address both financial and mental health outcomes.

**Objective:**

This scoping review systematically maps the research on money and mental health, examining: (1) the financial variables and data sources used, (2) modeling methods employed, and (3) methodological gaps that novel objective data might address.

**Methods:**

We systematically searched PubMed, PsycINFO, IEEE Xplore, ACM Digital Library, and Scopus. Papers were screened against predefined inclusion/exclusion criteria, and data extracted using a standardized spreadsheet. Analysis employed deductive coding guided by our research questions, refined iteratively through engagement with the data. PRISMA Extension for Scoping Reviews (PRISMA-ScR) was followed for reporting.

**Results:**

Of the 43 included studies, most (*n* = 34, 79%) examined mental health in connection with financial factors such as financial difficulty or financial strain, while a small number focused on predictive modeling with financial behavioral data (*n* = 5, 12%), macroeconomic indicators (*n* = 2, 5%), or intergenerational support between parent and child (*n* = 2, 5%). Depression was the most common outcome (*n* = 24, 56%), followed by anxiety, psychological distress, and bipolar disorder. Statistical methods dominated (77%), with 19% employing machine learning or deep learning. Ground truths relied predominantly on self-reported questionnaires—only four studies used objective financial data (three gambling records, one bank transaction).

**Conclusion:**

This review reinforces the complex, bidirectional relationship between financial circumstances and mental health. Most studies examined how financial difficulty affects mental health, while only a few explored how mental illness influences financial behavior, indicating a clear research gap. There is substantial opportunity to use objective financial data and more diverse analytical methods, particularly machine learning, to deepen understanding of the relationship and interactions between money and mental health and inform targeted interventions.

## 1 Introduction

Mental disorders are characterized by clinically significant disturbances in cognition, emotional regulation, or behavior that impair functioning and quality of life (1). According to the WHO, about 1.1 billion people—roughly 13–14% of the global population—were living with a mental disorder in 2021 (2). Beyond the psychological and functional burden, mental disorders are strongly associated with adverse financial outcomes. People with mental health conditions in the Organization for Economic Co-operation and Development (OECD) countries experience employment rates approximately 20 percentage points below those of the general population (3). Financial problems are associated with increased risk of symptom relapse (4) which may lead to greater psychiatric service utilization. Therefore, the relationship between mental health and financial circumstances is bidirectional (5, 6): individuals experiencing debt have more than threefold higher odds of having a mental disorder compared to those without debt (7) and people who are over-indebted have been shown to experience higher depressive symptoms (8). Accordingly, poverty is a key social determinant of mental illness, and evidence supports a causal relationship between poverty and mental illness (9).

This bidirectional relationship manifests across a range of mental health conditions. Bipolar disorder (BD) provides a particularly instructive example: manic episodes frequently lead to financial difficulties through impulsive and compulsive spending (10), with sustained negative impacts on wellbeing for years afterward. In a population research study of Bipolar 1 Disorder (BP1) and Schizophrenia patients, those with BP1 were 50% more likely to experience financially disruptive life events (11). Financial difficulties are also common in ADHD (12), and neurological disorders like dementia (13). More broadly, mental illness often impairs employment stability (14), increasing risk of income loss and financial difficulty. Conversely, financial hardship can heighten psychological stress, increasing vulnerability to mental illness. In the general population, a similar relationship is observed between money and overall wellbeing: financial wellbeing—the subjective assessment of one’s capacity to sustain present and future living standards while attaining financial independence (15)—is a critical determinant of overall personal wellbeing (16). Individual financial wellbeing is shaped by financial attitudes, literacy, psychological dispositions, financial behaviors such as compulsive buying and demographic characteristics (17), alongside macroeconomic variables such as employment, interest and inflation rates (15). These findings align with behavioral finance research demonstrating that psychological states, cognitive biases, emotional regulation, and risk perception substantially influence financial decision-making and financial behaviors (18). Financial satisfaction (17, 19) and financial wellness (19) represent conceptually-related constructs that are often used interchangeably with financial wellbeing. While financial wellbeing is inherently subjective, researchers increasingly operationalize it using both objective indicators—such as income, financial life-cycle stage, and financial management behaviors—and subjective assessments (e.g., perceived income sufficiency) (20).

Digital phenotyping research has primarily focused on the use of smartphone sensing data and wearable devices as digital markers of human behavior (21) and psychopathological states (22). However, several challenges associated with these data sources have been reported, including inaccurate readings (e.g., a phone left on a table for extended periods being misinterpreted as inactivity or sleep) (23), data loss or missing data due to poor user adherence (24), battery drain, the need for sustained user engagement, and concerns regarding data quality (25). Additionally, many longitudinal digital phenotyping studies rely on repeated follow-up assessments (26), which may become increasingly difficult to sustain over time.

The rapid adoption of digital payment methods (27, 28) is generating an increasingly rich source of objective financial behavioral data. As individuals and businesses shift from cash to digital transactions and banking infrastructures, open banking APIs (29) now enable access to fine-grained, timestamped records of income, expenditure, and other financial behaviors. These data, which are readily available through banking systems, can be retrospectively and prospectively collected with minimal participants burden, presenting a potential alternative or complement to smartphone-derived data. Preliminary work has demonstrated the feasibility of using objective financial data—including bank transaction records—for mental health monitoring (30). This creates an emerging opportunity for passive, real-time behavioral monitoring that could complement traditional self-report approaches and enable the development of personalized financial management interventions to improve mental health outcomes.

Despite the well-established relationship between money and mental health, no review has systematically synthesized the full range of financial variables studied, the data sources used to measure them, or the analytical methods employed. Existing reviews have addressed specific financial constructs such as debt (7) and financial wellbeing (31) in relation to mental health, but none has mapped the broader landscape of financial variables in this field. A recent review (31) found that existing studies predominantly rely on subjective financial measures and offered recommendations focused on public health policy, financial literacy, and educational interventions, with limited attention to individual-level behavioral patterns that could be captured through objective data. Furthermore, heterogeneous measurement approaches for financial variables limit cross-study comparisons. While the relationship between money and mental health is shaped by several multi-dimensional factors—including economic conditions such as socioeconomic status and employment stability, negative financial effects such as debt burden, and sociodemographic variables, e.g., education and physical health (6)—the absence of a methodological synthesis leaves researchers without a structured foundation for leveraging objective financial data for mental health assessment and intervention development.

To address this gap, this scoping review systematically maps the mental health conditions examined, financial variables studied, data sources used, and analytical methods employed across the existing literature. By synthesizing measurement approaches, data sources, and analytical techniques, this review provides a structured foundation for integrating objective, fine-grained financial behavioral data into future study designs and for advancing the nascent field of financial phenotyping—that is, the use of objective financial behavior data to identify patterns relevant to health and wellbeing.

The research questions underlying the review are:

1. What financial and mental health variables have been examined?
2. What data sources have been used to measure financial factors and mental health?
3. What analytical approaches have been used to examine mental health-finance relationships?

## 2 Methods

### 2.1 Protocol

This review examined research focused on money and mental health, and the analytical methods used. To standardize the review process and facilitate reliability and reporting of results, the PRISMA-ScR (PRISMA extension for scoping review), a standardized method and checklist for conducting scoping reviews was applied (32).

### 2.2 Information sources

This study explored three key themes: mental health, finance, and modeling (statistical analysis or machine learning). To ensure comprehensive coverage, we selected five relevant databases, including health related databases such as PubMed and PsycINFO, information technology (IT) databases including IEEE Xplore and ACM Digital Library, and finally Scopus, which provides broad interdisciplinary literature relevant to the scope of the review. We selected a combination of these databases to maximize coverage of literature relevant to the review objectives while minimizing unnecessary overlap in indexed records. Pilot searches were conducted across the selected databases, and the search strings were iteratively refined to optimize retrieval of studies relevant to the three domains of interest. Search keywords were developed through a systematic and iterative process. Initial terms were identified from a closely related ongoing study (33) and from keywords used in other papers related to mental health (30, 31). This process was further informed by consultation with a University College Dublin librarian experienced in systematic and scoping review methodology, who provided guidance on search string construction and database-specific syntax. Relevant MeSH (Medical Subject Headings) terms, such as “statistical model,” were also used to develop the search strategy to align with controlled vocabulary used in health science databases and improve retrieval sensitivity. The resulting search strings were piloted across the selected databases and iteratively refined to optimize coverage across the three domains of interest: mental health, finance, and analytical or modeling approaches. Preliminary search outputs were reviewed to identify irrelevant retrieval patterns and gaps in coverage, leading to modifications of keywords and search combinations where necessary. The final search strategy was validated by confirming the retrieval of key studies in the field. Reflecting the aims of the review, the keywords captured three key areas as presented in Table 1. Given the focus of this review on analytical approaches, modeling-related terms were included in the search strategy and eligibility criteria to ensure the review captured studies employing statistical or machine learning methods.

**Table 1:** Search keywords highlight themes in mental health, modeling and finance.

Theme	Search terms
Mental health	“mental health” OR “mood disorder” OR “psychological distress” OR “eating disorder” OR “anxiety” OR depress* OR “OCD” OR “bipolar disorder” OR “schizophrenia” OR “substance abuse” OR “emotional wellbeing”
Modeling	“financial technolog*” OR “fintech” OR “statistical model*” OR “machine learning” OR “data science” OR “artificial intelligence” OR “AI” OR “data analytics”
Finance	financ* OR bank* OR money OR gambling

This table presents the search keywords grouped into three thematic domains—mental health, modeling approaches, and finance—to support identification of interdisciplinary studies examining relationships between financial factors, mental health variables, and analytical methods.

### 2.3 Eligibility criteria

To be included, the study had to be (1) peer-reviewed, (2) address a mental health condition as defined by DSM or ICD criteria or associated mental conditions such as distress, mental health and wellbeing (3) explore financial behavior or the relationship between finance and mental health conditions, (4) be written in English. The exclusion criteria specify that studies focused on the financial behavior or data of non-human entities, such as governments or companies, were not eligible. Additionally, studies that did not employ modeling approaches to examine the relationship between mental health and financial factors were excluded to maintain focus on analytical methods. Although mental illness is identified as a risk factor for suicide (34), we excluded results that measure the relationship between finance and suicide to limit the scope of our results to the association of mental health and money, rather than the wider potential consequences of mental ill health.

### 2.4 Selection of sources of evidence

**Figure 1:**
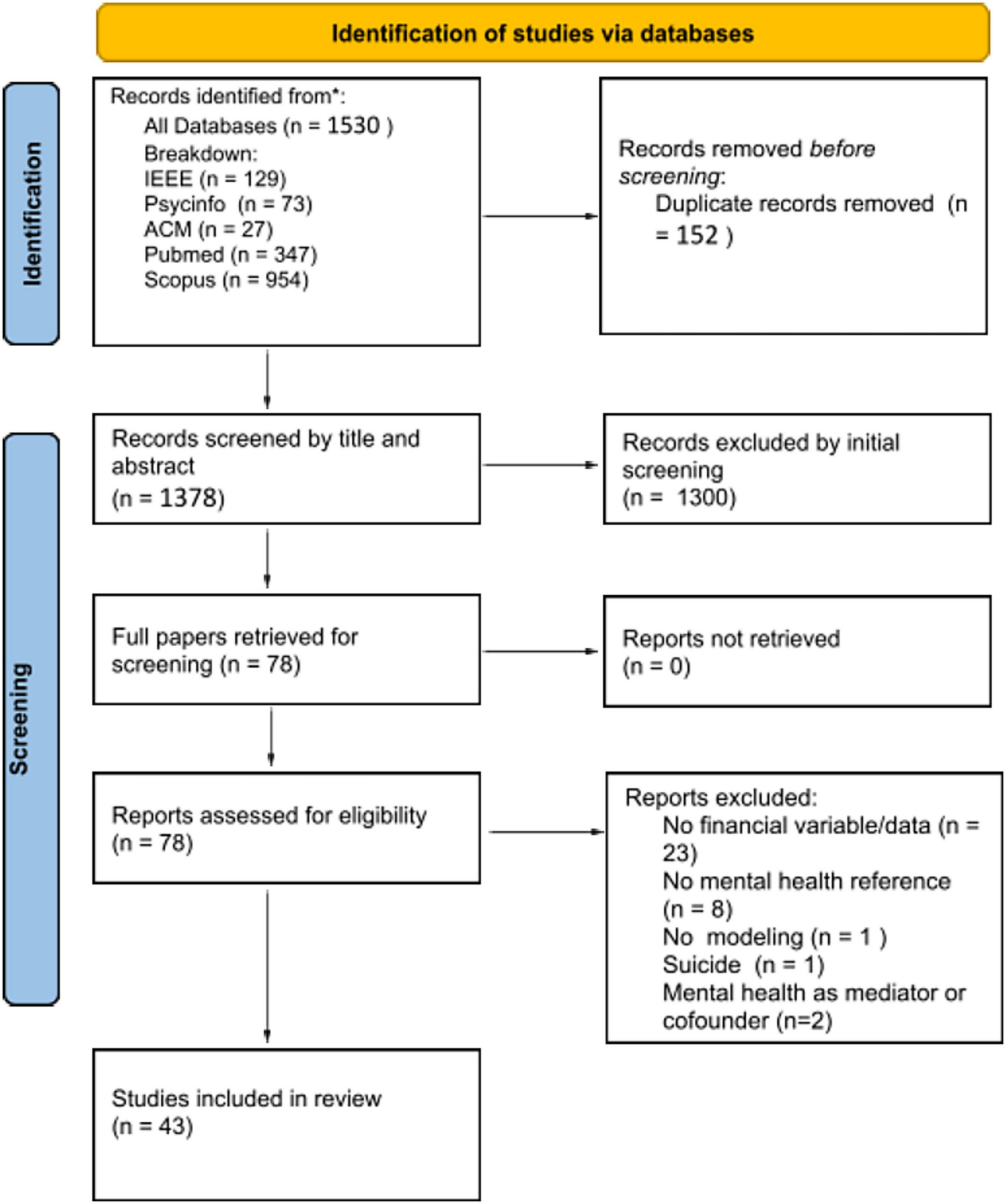
PRISMA-SCR flow chart.

Rayyan (35), a research tool for conducting systematic reviews, was used to conduct this scoping review. The first author extracted the title and abstracts of the results to Rayyan in May 2024. The initial search returned 1,530 papers of which 152 were duplicates and filtered out on Rayyan. Following the initial screening, 1,300 records were excluded for not meeting the scope of the review based on the eligibility criteria. The most common reason for exclusion was failure to meet the criterion requiring studies to explore financial behavior or the relationship between finance and mental health conditions. Many retrieved records focused exclusively on mental health or financial outcomes in isolation, rather than examining their relationship. This also included studies that focused primarily on governmental or business finance rather than individual financial behavior, and studies involving non-human subjects. This screening process highlighted the limited availability of studies directly aligned with the objectives of the review. Screening was conducted iteratively based on the inclusion criteria applied to titles and abstracts with the first author screening all records and the second author independently screening a random 50% sample. Following screening, both authors reached agreement on the included studies, with 27 papers initially identified as conflicts. A third author validated the screening process and supported the resolution of any disagreements. Deliberations were conducted on papers designated as “maybe” or those conflicted by the other authors until consensus was reached. Conflicts in study selection were discussed and resolved by both authors, which led to further refinement of the eligibility criteria. Initially, studies directly examining the relationship between financial behavior and mental health were prioritized. Following these discussions, the eligibility criteria were expanded to include studies assessing financial difficulty and socioeconomic determinants of mental health where these aligned with the objectives of the review. This resulted in 78 papers being retained for full-text screening by both authors, of which 43 studies were included in the final scoping review, reflecting reduction ratios similar to those reported in reviews using comparable databases (36). Figure 1 shows the flow chart for the selection of articles.

### 2.5 Extracting, charting, summarizing and reporting results

From the fully read papers, the first author extracted the following data (i) year of publication, (ii) title, (iii) location, (iv) study type, (v) summary, (vi) data types, (vii) financial features, and (viii) modeling techniques and others into a standardized spreadsheet. The categories of the research were extracted through a deductive method based on research questions guided by predefined keywords (37) and refined based on feedback from the second, third and fifth author who supervised the data extraction process. We organized codes into three categories: (i) mental-health variables; (ii) financial variables (e.g., financial difficulty, financial behavior, economic indicators); and (iii) modeling approaches.

## 3 Results

### 3.1 Overview of study characteristics

Our results show that there is growing research interest in mental health and finance, with most work (i.e., 34 papers, 79%) in our review published between 2015 and 2024. To gain a general overview, we categorized the studies by their study type. Most of the studies (*n* = 27, 63%) were cross-sectional, 16% (*n* = 7) were longitudinal, 7% (*n* = 3) were observational and 7% (*n* = 3) were exploratory, 4.6% (*n* = 2) both cross-sectional and longitudinal, and 2.3% (*n* = 1) a conceptual study.

### 3.2 Mental health characteristics

The studies identified covered a range of topics that can be grouped into those focused on specific disorders or symptoms and those on non-specific broad mental health terms like distress, wellbeing. The majority of the studies considered depression or depressive symptoms. An overview of specific conditions reported are: depression (*n* = 24, 56%), anxiety (*n* = 4, 9%), bipolar disorder (*n* = 3, 7%), and general non-specific mental health terms such as psychological distress (*n* = 4, 9%), psychological and subjective well-being (*n* = 4, 9%), online shopping addiction (*n* = 1, 2%), problematic gambling (*n* = 3, 7%), ADHD (*n* = 1, 2.3%), self-reported mental health (*n* = 3, 7%). Some of the studies, (*n* = 12, 28%), considered more than one mental health theme, e.g., anxiety and depression.

### 3.3 Modeling mental health assessment based on financial and socioeconomic variables

We identified considerable variation in analytical approaches across the 43 included studies. The association between MH and money was assessed predominantly through modeling techniques such as statistical analysis and machine learning. The majority (*n* = 33, 77%) employed statistical analysis while (*n* = 8, 19%) used machine learning (ML), 1 applied both statistical and ML methods, and 1 study proposed a conceptual framework with macroeconomic variables (i.e., no experimentation but modeling conceptual). The models explored were dependent on the task. For example, regression analysis for understanding associations between variables (38) supervised prediction for regression or classification (39) and unsupervised learning for anomaly detection (30). Most studies (*n* = 32, 74%) reported a single modeling technique, while (*n* = 11, 26%) explored multiple approaches. Rather than cataloging each method individually, we synthesize key patterns and methodological insights to guide future research design and we provide the modeling details in Table 2.

**Table 2:** Summary of the financial variables and analytical methods employed.

Mental health condition	Ground truth	Financial variables	Analytical technique	Relationship between mental health and financial variables
ADHD (*n* = 1)	Development and Wellbeing Assessment (DAWBA) (50)	Socioeconomic status measured by parental income, education, employment, marital status, maternal age at birth, housing tenure, large family size, financial difficulties (50).	Multivariable regression (50), Logistic Regression (50)	More families of children with ADHD reported financial difficulty (50) Emergence of ADHD twice as likely in children whose families are perceived to have financial difficulty (50). Financial difficulties a more accurate predictor of ADHD, both directly and by mediator effects.
Behavioral addiction (*n* = 4)	Questionnaire for Online Shopping Addiction (45), Problem Gambling Severity Index (PGSI) and raw gambling data from site (43, 44), Pathological Gambling and Epidemiology [PAGE] (39)	Frequency (43, 44), Variability (44), Maximum, Most bets in a day (44), Repeat engagement on site (44), Average (and SD) of weekly bet (44), age (43, 44), amount of money deposited (43), amount of money bet (43), number of gambling days (43), average monetary loss per gambling day (43), average monetary loss per session (43), average number of monetary deposits per session (43), account depletion (43), number of play breaks (43), active gambling days (39), maximum amount of money lost within 1 year (39), discounts and offers (45), buying non essential products (45)	Multilayer Perceptron (MLP) (45), Support Vector Machine (SVM) (44, 45), Naive Bayes (45), Decision Tree (44, 45), Random Forest (43, 44, 45), K-Nearest Neighbor (KNN) (44) Logistic Regression (44), Artificial Neural Network (ANN) (44), Gradient Boost Machine Learning (GBML) (43), K-Means Clustering (43), Logistic regression (39)	RF achieved a test AUC value of 0.729 for problem gambling (43), AUC value of 84.33% (44), Neural Network achieved an F1-Score of 88 (45) for online shopping addiction
Bipolar disorder (*n* = 3)	Bank Statements and National Institute of Mental Health Life Chart Method (30), Data from National Epidemiologic Survey on Alcohol and Related Conditions(NESARC). Utilized the Alcohol Use Disorder and Associated Disabilities Interview Schedule-IV (AUDADIS) (62), Mood Disorder Questionnaire (MDQ) (72), Massively Multiplayer Online Game (MMOG) (72).	Transaction volume (30), frequency of expenditure (30), burstiness (30), economic problems (major financial crisis, declared bankruptcy, more than once been unable to pay bills on time, household income less than 150% of the federal poverty threshold, received public assistance) (62), gacha or purchase behavior (72)	One way Welch ANOVA (30), Games-Howell (30), Isolation Forest (30), Logistic Regression (62), Transformer-Variational Autoencoder (t-VAE) (72)	Expenditure spikes in periods of moderate manic and mild manic symptoms (30), Interpersonal instability and financial hardship associated with significantly higher risk of incident and recurrent mania (62), Financial/interpersonal instability and economic difficulty associated with depressive episodes (62), manic/hypomanic episode duration was likely to produce higher frequency of gacha and purchase behaviors than non-manic episode duration (72).
Mental/psychological distress (*n* = 4)	Kessler index (51, 66), Administration of Occupational Safety and Health (AOSH) (94)	Economic indicators such as Currency Exchange, Unemployment rate etc. (73), Internal measures of mental distress such as salary structure, sector/industry, work-family life satisfaction (73), Subjective Financial Strain (66), Low Income (66), Job downsizing (94), financial stressors (financial worries, healthcare insecurity and food insecurity) (51)	Data Mining (Conceptual) (73), Big Data (Conceptual) (73), Random Effects (66), Fixed Effects (66), Ordinary least squares (OLS) regression (94), PCA with varimax rotation (51), Multivariable regression (51),	Low-Income Measure Threshold Positively Associated with Distress (66), Mastery Measured by Pearlin and Schooler Mastery Index Negatively Associated with Distress (66), Working in a downsized department most strongly related to psychological distress (94), Each financial stressor adversely associated with Serious Psychological Distress (SPD) both individually and Cumulatively (51).
Depression/depressive symptoms (*n* = 26)	Patient Health Questionnaire (PHQ-9) (47, 52, 69, 83, 95), PHQ-2 (59), Center for Epidemiologic Studies Depression Scale-10 items (CESD-10 (54, 65, 77), CES-D-11 item (55), CES-D (49, 61, 68), Geriatric Depression Scale (GDS) (38, 53, 57, 84), ICD-10 codes (40, 84), Edinburg Postnatal Depression Scale (EPDS) (46, 56), Mini-international Neuropsychiatric Interview (MINI) 5.0 (58), Depression Anxiety and Stress Scale(DASS 21) (60), Whooley Depression Screen (96)	Economic factors such household income, consumption, debt etc. (52), Family/household income (47, 49), financial problems (47), material hardship (related to food, housing and health) (53), Subjective Household Financial Strain (54), Subjective Household Food Insufficiency (54), Economic adversity (household income, financial strain, measure of neighborhood-level poverty) (55), savings (40), socioeconomic data, e.g., income, employment status, debt worries (56), Financial Hardship(difficulty paying bills, food insecurity and reduced medication use due to cost) (61, 68), Income less than threshold (38), Income-based poverty (57), financial strain (57), intergenerational financial and instrumental support (77), financial problem (69), self-rated financial status (58), perceived financial status (95), financial difficulties (59), Financial Worries (49, 96), Financial Constraint (83), Financial State (46)	Univariate regression (52) Multivariable regression (52), Mediator Model (65), ANOVA test (47, 52, 96), t- test (57, 83), Binary Logistic Regression (53, 57, 61, 77, 95), Multiple logistic regression (59, 60, 84, 96) with backward stepwise (58), Bootstrapping (52, 53), chi statistics (53, 54, 57, 83, 96), Regression Analysis (54), Hierarchical Linear Modeling (55), Multiple Linear Regression (40), OLS (56), Multivariate Ordinal Logistic Regression (38), Multivariate Logistic Regression (83), Multivariate Generalized Linear Mixed Modeling (GLMM) (95), Gradient Boosting Machines (47), Distributed Random Forests (47), Extreme Randomized Forests (47), Random Forest (46), Naive Bayes (46, 47), Support Vector Machine (46, 47), Multilayers Neural Network(MNN) (47), Decision Tree (46, 47), XGBoost (49), K-Nearest Neighbor(KNN) (46), AdaBoost (46)	Presence of household debt, capacity to pay, household consumption associated with depression severity (52), Mediation effects between stress and depressive symptoms in the relationship between financial strain and self-rated (physical) health (65), food insecurity associated with depressive symptoms (53), Financial strain associated with depressive symptoms in crude and adjusted models (54), lower household income, higher financial strain and lower neighborhood SES associated with higher numbers of depressive symptoms (55), Early retirement due to mental health problems associated with reduced retirement savings (40), There is a relationship between financial hardship and depressed mood in mothers of infants (56), those who experienced financial hardship more likely to report three or more depressive symptoms (61), Income less than threshold (Bahraini Dinar (BD) of 200 (≈£377)) associated with higher risk of depressive scores (38), − Social exclusion, income poverty and financial strain are positively associated with depressive symptoms, but only social exclusion and financial strain maintain their significance after all covariates are adjusted (57), Those receiving financial support, receiving instrumental support (a form of social support), and receiving and providing financial and emotional support had 19, 14, 23, and 24% lower odds of depression (77), people who reported financial problem or health concern as their main source of stress more likely to experience depressive mood (69), poor financial status significantly associated with depressive disorders (58), financial and employment-related difficulties are important risk factors for depression (95), late-life depression significantly associated with financial difficulties (84), financial difficulties marginally significant with depression (59), students in financial difficulty more likely than those without to experience depressive symptoms (60), financial worries strongest association factor for depressive symptoms (96), financial constraint one of the strongest predictors of depression (83), financial state one of the important risk factors for prenatal depression (46).
Anxiety(*n* = 4)	Generalized Anxiety Disorder (GAD-7) (47, 49), GAD-2 (59), Depression Anxiety and Stress Scale (DASS 21) (60)	Family/household income (47, 49), financial problems (47), Financial worry (49), Financial Difficulties (59, 60)	Logistic Regression (59, 60), Gradient Boosting Machines (47), Distributed Random Forests (47), Extreme Randomized Forests (47), Naive Bayes (47), Support Vector Machine (47), Multilayers Neural Network(MNN) (47), Decision Tree (47), XGBoost (49)	Financial difficulties/problems statistically associated with anxiety (59, 60).
Mental health (*n* = 7)	Psychic morbidity measured by Goldberg Health Questionnaire (74), Perceived Stress Scale-4 (65), SF-36 (76), Psychological distress measured by Kessler 10 (76), ICD-10 codes (40), General Social Survey (42), CES-D12 (63), CES-D-20 (64), General Social Survey (42)	Macroeconomic variables, e.g., lower healthcare spending per capita, higher percentage of temporary workers (74), Financial Strain (65) household income (65, 76), annual household income (76), individual deprivation Index (76), financial unhealthy (failure to pay one bill or unable to save) (42), child related educational debt (63), financial status (64)	Multilevel logistic regression (74), Logistic Regression (64), Chi-square (64, 74), Fixed effects regression (76), OLS (42), Stepwise Forward Regression (42), Ordered Logit Estimation (42), Weighted Linear Regression (63), Multiple Regression (40, 65)	Socioeconomic variables such as risk of poverty, income per capita per household, GDP, employment rate not found to be linked to worse mental health (74), financial strain positively correlated with increased psychological stress (65), decreased mental health and increased psychological distress for those that moved into inactive labor force, individual deprivation and low household income (76), financial unhealthiness predicts 7% lower likelihood of self-rating of excellent mental health (42), Any child-related educational debt associated with fewer depressive symptoms with father (63), no significant relationship between financial status and depressive symptoms (64), Mental health problems lead to lifetime economic and social disadvantage (40).
Psychological/subjective/mental well-being (*n* = 4)	CESD-10 and life satisfaction (75), World Health Organization Quality of Life Survey (WHOQOL-100) (67), CASP-12 (48), WHO-5 (97)	Financial exchange through financial aid (75), poverty status based on monthly income level (75), economic well-being based on annual household income (75), working poverty(financial insufficiency, restricted standard of living) (67), financial variables such as number of owned cars, current job status, difficulty making ends meets, etc. (48), perceived financial strain (97),	Sensitivity analyses (75), Ordered logistic Regression (75), Binomial Logistic regression (75), Linear regression (75), Logistic Regression (48, 67), Generalized Estimating Equation (67), Logistic Regression with Lasso (48), Random Forest (48), Multilevel Linear Regression (97)	Older adults who give financial support less likely to be depressed and reported higher levels of life satisfaction (75), effect of receiving financial support not significant for depression, but related to higher levels of satisfaction (75), psychological well-being of both sexes significantly influenced by the experience of working poverty(financial deficiency and restricted standard of living) (67), socioeconomic inequality in mental well-being (i.e., lower socioeconomic status had poorer mental health) 40% narrower among respondents reporting good access to green/recreational areas, compared with those with poorer access (97).
Post-traumatic stress disorder (*n* = 1)	Impact of Event Scale-Revised (IES-R) (70)	Financial sufficiency to make ends meet (70)	Multivariate Logistic Regression (70)	Financial insufficiency increased family member’s risk of significant PTSD symptoms (70)

This table summarizes the mental health conditions examined across the included studies, the corresponding ground-truth assessment tools, financial variables analyzed, and the statistical or machine learning methods employed. The table demonstrates substantial heterogeneity in the measurement of both financial and mental health constructs, with depression representing the most extensively studied variable. Financial difficulty, e.g., hardship, strain, emerged as the most frequently examined financial variables, while multivariable regression and logistic regression were the most commonly applied analytical techniques. Machine learning approaches were primarily observed in studies on behavioral addiction, depression, anxiety, and wellbeing. Overall, the table highlights the multidisciplinary nature of the field and the growing use of computational methods to investigate relationships between financial behavior and mental health outcomes.

### 3.4 Aligning methods with research goals

Analytical methods used were understandably closely aligned with research objectives across studies. Statistical methods dominated among the cross-sectional studies (i.e., 93%). The majority of studies (*n* = 27; 63%) examined associations between financial variables—such as income, financial strain, and socioeconomic status—and mental health outcomes. In contrast, only two studies assessed the relationship between mental health and subsequent financial outcomes [e.g., savings and income (40), and employment status (41)], and a study (42) investigated the bidirectional relationship between financial difficulty (i.e., unhealthiness) and mental health. To examine the distribution of data and identify missing data, descriptive statistics was employed in some (*n* = 13, 30%) of the studies. The studies tested theoretical models and quantified relationships, with logistic regression (*n* = 19, 44%) and linear regression (*n* = 13, 30%) being the most common techniques for assessing how financial difficulty is associated with depression and anxiety symptoms.

In contrast, all studies with exploratory, or predictive aims employed ML approaches. These studies sought to identify at-risk individuals or detect behavioral patterns including problem gambling (43, 44), online shopping addiction (45) and manic spending in bipolar disorder (30). Table 2 summarizes the analytical methods employed in the identified papers.

### 3.5 Machine learning applications: performance and interpretability trade-offs

Among the 8 ML studies, most (i.e., 6, 75%) used supervised learning models to predict mental health outcomes, while (*n* = 2, 5%) employed unsupervised methods - clustering with K-Means Clustering in Auer & Griffiths (43) to identify high-risk gambling patterns and anomaly detection using isolation forest (30) to flag unusual spending periods in bipolar disorder. These methods were explored to identify behavioral patterns in player tracking features on a gambling site and spending transaction data, respectively. The cluster analysis was also used to identify which variable was associated with higher or lower likelihood of self-reported gambling.

Reported ML model performance varied but was generally strong, with performance metrics including accuracy (44), area under the curve AUC (43), F1 score (45), sensitivity (46), positive predictive value (46), negative predictive value (46), and Mathew’s Correlation Coefficient (47).

While most studies explored logistic regression for statistical analysis, 2 studies employed logistic regression for classification. Ensemble methods—which combine multiple models to improve predictions—showed consistently strong performance across studies. Random forest models achieved notable success in predicting problem gambling (>80% accuracy) (44), online shopping addiction (45), and subjective well-being (48). In head-to-head comparisons, random forests outperformed both traditional logistic regression (48) and other ML approaches like gradient boosting (43) in some specific studies and tasks. XGBoost, AdaBoost, and Gradient Boosting Machine Learning were similarly effective in predicting depression and anxiety symptoms in perinatal populations using sociodemographic and financial variables (46, 47, 49), with GBML achieving best performance in one multi-model comparison (47). Support Vector Machines (SVM) also gave comparative performance in minimizing misclassification error in detecting online shopping addiction using questionnaire data (45). Model performance across different tasks varied considerably depending on how outcomes were defined and measured. For instance, while random forest achieved over 80% accuracy for problem gambling prediction (44), the area under the precision-recall curve ranged from only 38–58% depending on the Problem Gambling Severity Index threshold used.

### 3.6 Relationship between mental health and financial variables

Findings from a significant proportion of the papers show a bidirectional relationship between mental health and finance (5), where mental health challenges can lead to financial difficulty, while financial difficulty is associated with mental health symptoms. For example, in Russell et al. (50), families of children with ADHD reported financial difficulty, and the emergence of ADHD is twice as likely in children whose families have financial difficulty. Financial stressors in Tsuchiya et al. (51) are adversely associated with Serious Psychological Distress (SPD) both individually and cumulatively. Some studies have shown that the presence of household debt (52), food insecurity (53), financial strain (54, 55), financial hardship (56), income poverty (57), poor financial status (58), are associated with depression/depressive symptoms. Financial difficulties are statistically associated with anxiety (59, 60). Marshall et al. (61) assessed the relationship between financial hardship and depression and discovered that those who experience financial hardship are more likely to report depressive symptoms. Gilman et al. (62) examined the increased risk of both initial and recurrent manic episodes associated with financial hardship and found that participants with financial or economic difficulties were more likely to have a recurrent manic episode.

Interestingly, some studies observed a contrasting relationship between debt, financial status and depression/depressive symptoms. The presence of any debt accrued from a child’s education was associated with fewer depressive symptoms with the father (63). This was attributed to the father having a positive outlook about being able to carry out their role of supporting their child. In another study (64), there was no significant relationship observed between financial status (i.e., access to scholarships) and depressive symptoms, highlighting the heterogeneity of financial measures used in this field and the variability in their statistical associations with mental health outcomes. Early retirement due to mental health problems was associated with reduced retirement savings (40), suggesting the effect of mental health challenges on finances. Sociodemographic variables—including gender, age, race, education, income, and marital status—were commonly included as covariates. In some studies (*n* = 10, 23%), additional controls such as self-rated health and prior mental health conditions [e.g., previous depression (56) or diagnosed disorder (62)] were observed when examining the relationship between financial variables and mental health outcomes.

### 3.7 Types of financial variables studied

Our findings show that financial variables that have been used in the studies identified in this scoping review can be broadly categorized into: (1) financial difficulties such as income inadequacy or difficulty paying bills, (2) financial behavior like manic/hypomanic spending or gambling, and (3) society-level economic indicators such as GDP, inflation etc.

### 3.8 Defining and measuring financial difficulties

Financial difficulty was described using different variables across studies, and our review identified substantial heterogeneity in how financial difficulties were defined and measured. Although these variables differ, they similarly emphasize the negative aspects of financial difficulty, particularly the inadequacy of economic resources and the inability to afford basic needs. For example, Savoy et al. (65) related financial income inadequacy to a condition where people have a negative subjective perception of the amount of income they have, compared to their needs. This was also described as financial strain in some studies and was measured based on whether individuals had sufficient money or income to meet daily needs (57, 66). Financial difficulty can also arise when spending exceeds income (42) and the effect of this strain in a household is more pronounced on the primary income provider (54).

The concept of working poverty, in Vetter et al. (67), extends the idea of income inadequacy (also referred to as financial deficiency) by combining insufficient income with a restricted standard of living. Related constructs include lack of economic resources (57), and financial hardship, defined in Marshall et al. (61) as the inability to meet life obligations with limited economic resources. In Johnson et al. (53), hardship is described as material hardship, occurring when individuals cannot afford basic needs such as food, housing, and medical care. This aligns with the concept of material deprivation in Vetter et al. (67) where individuals lack two or more of 10 essential items—such as adequate home comfort, holidays, a car, a color television, or dental care—considered necessary in a given population. Other indicators of economic strain include bankruptcy, inability to pay bills on time, household income below 150% of the federal poverty threshold, and reliance on public assistance (62). Financial deficiency, in Vetter et al. (67), was defined by household income of less than 60% of the weighted OECD median household income; however, Marshall et al. (61) identified extending hardship measures beyond traditional socioeconomic indicators (e.g., income and education) to include perceived income adequacy, and ability to meet basic needs such as food, shelter, transportation costs and medical needs. Other approaches included household income, parent-reported financial strain and neighborhood-level poverty to capture economic adversity (55).

Overall, the literature refers to various overlapping forms of financial difficulty, including material hardship (53), working poverty (67), financial hardship (58, 68), financial unhealthiness (42), financial problems (69), financial insufficiency (70), economic adversity (55), or economic problems (62). Related variables often associated with financial difficulty include socioeconomic status (50, 58, 60), income poverty (57, 66, 67), and inability to pay bills (42, 61, 68). Table 2 summarizes the different variables used to represent financial difficulties, which were mostly examined as predictors of mental health.

### 3.9 Financial behaviors

Prior research has shown that poor mental health leads to poor financial decisions or behaviors (71), which in turn worsen mental health (5). In this review, only 5 (11%) of the studies considered financial behavior such as online shopping addiction (45), bipolar spending (30), and problematic gambling behavior (39, 43, 44). These studies suggest that financial behaviors can be characterized by temporal patterns (frequency, timing), magnitude (transaction amounts), and behavioral signatures (e.g., gambling patterns, impulse purchase clusters). For example, purchase behavior is shown to increase during manic/hypomanic episodes for those with bipolar disorder (72). Table 3 summarizes financial behavioral variables examined across studies, illustrating the range of behaviors potentially relevant for mental health assessment.

**Table 3:** Financial behavioral categories and variables.

Behavioral category	Variables
Spending frequency	Frequency of expenditure (30), online shopping frequency (45)
Gambling patterns	Maximum amount lost in gambling (39), repeat engagement in gambling(number of times per week three or more deposits made in 12 h window) (44),
Spending category	Non-essential purchases (45), purchase behavior (72)
Spending magnitude	Deposits and expenditures (44), transaction volume (30), trough or minimum amount of spending (44), crest (maximum) of spending (39)
Financial interactions	Financial exchange/support direction (e.g., child to parent and vice versa) (75, 77)
Temporal or statistical measures	Burstiness (to measure stability and randomness) (30), and variability (43, 44).

This table categorizes the financial behavioral variables identified across the reviewed studies into key domains, presenting potential features researchers can investigate in future studies to examine relationships between financial and mental health variables.

### 3.10 Economic indicators

In addition to individual-level financial spending or behavioral variables, some studies also examined national-level economic indicators. For example, Mehreen et al. (73) proposed a conceptual framework in which factors such as currency exchange rates, unemployment, GDP ratio, balance of payments deficit, oil prices, gold reserves, per capita income, and foreign diplomacy were considered potential predictors of mental distress. In an empirical analysis, Ruiz-Pérez et al. (74) found that indicators such as GDP per capita, employment rate, risk of poverty, and household per capita income were not associated with worse mental health (measured via psychic morbidity). However, higher healthcare spending per capita and a greater proportion of temporary workers were linked to poorer mental health outcomes.

### 3.11 Financial ground truth

Financial ground truths vary from subjective self-report data such as questionnaires and interview data, to objective data such as data from gambling platforms (*n* = 3, 7%) and spending data (*n* = 1). All studies examining financial difficulties relied on self-report or interview data. Common ground truth included validated instruments such as the Financial Strain Questionnaire (65), Likert-style scales (57, 66, 68), dichotomous variables of yes/no (42, 47, 55, 61). Large-scale regional survey datasets were also used, including the Health and Retirement Study (HRS) (61), Korean Longitudinal Study of Aging (KLoSA) (75), the Survey of Family, Income and Employment by Statistics New Zealand (NZ) (76), the China Health and Retirement Longitudinal Study (CHARLS) (77), and an adaption of Established Populations Epidemiologic Study of the Elderly (54) among others.

The measurement of financial strain varied across studies. For example, Koltai et al. (66) assessed how often families lacked enough money for essentials (e.g., food, clothing), and their financial situation at the end of the month, while Lee and Chou (57) used a 5-point scale to rate income adequacy for daily needs. In Kingston (55), financial strain was measured by self-reported ability to afford basic needs over the past 6 months. Financial difficulty was sometimes captured as a binary variable indicating the inability to afford heating, clothing, rent/mortgage or other essentials (50). While some studies relied on a single dichotomous variable (50, 70), others used multiple measures such as difficulty paying bills, reducing medication use due to cost (68), and food insecurity (61).

Economic indicators at the national level were collected from sources such as the National Institute of Statistics, Eurostat and BBVA foundation in Ruiz-Pérez et al. (74). Financial behaviors were measured using both self-report instruments and objective transaction data. For instance, problematic gambling was assessed with tools including the gambling section of the WHO’s World Mental Health (WMH) Composite International Diagnostic Interview (CIDI) (39), the Pathological Gambling and Epidemiology (PAGE) (39), the Problem Gambling Severity Index (PGSI) (44), alongside behavioral data from gambling platforms (43, 44). Online Shopping Addiction data was measured via online questionnaire in A. G. S N, kumara BTGS (45).

Only one study used objective bank transaction data to assess manic spending in bipolar disorder (30), analyzing a single participant over a 24-month period (3,373 transactions). Mental health episode labels were generated using the National Institute of Mental Health Life Chart Method, with input from family members and corroborating sources such as emails, photographs, and SMS logs.

## 4 Discussion

In this scoping review, we identified 43 studies that analyzed money and mental health, and documented the mental health and financial variables employed in their analyses. The analytical landscape is dominated by traditional statistical methods (77% of studies) seeking to assess hypotheses using regression analysis, with machine learning approaches representing only 19% of the studies. Across all studies, three categories of financial variables were used to investigate a range of mental health conditions: (1.) financial difficulties, (2.) financial behavior, and (3.) economic indicators. We found that there is significant research on statistical assessments of financial difficulty, strain, worry, insufficiency on mental health, with these variables largely being significant predictors of mental health, particularly depression. However, research on the connection between mental health and both financial behavior and societal-level economic factors was limited.

### 4.1 Limitations of cross-sectional research and opportunities for longitudinal financial phenotyping

The majority of the studies in this review are cross-sectional and do not establish causal evidence, showing consistency with previous similar reviews (7, 31). This likely reflects the relative ease of collecting cross-sectional data at one time point unlike longitudinal studies which collect data at multiple time points and are more demanding. Nevertheless, longitudinal studies enable researchers to assess temporal sequences between exposures and outcomes, thereby strengthening causal inference. Future studies should prioritize longitudinal designs using passively collected objective financial data to better establish temporal relationships and strengthen causal inference. Passively collected objective financial data (30), for example using the Plaid API, can capture data at multiple time points, thereby offering a promising low-burden approach to facilitate such studies.

### 4.2 Modeling trends, limitations, and future directions in machine learning approaches

Among the ML studies, the vast majority employed supervised learning for forecasting (48) or predicting (47, 49) mental health outcomes based on self-reported financial variables. This dominance likely reflects the design of research goals based on possible availability of mental health labels combined with the difficulty of obtaining actual financial transaction data.

This narrow methodological focus reveals several untapped opportunities. First, unsupervised learning methods—such as clustering algorithms—could help identify latent patterns in financial behavior associated with mental illness without requiring predefined outcome labels. Second, no studies applied time-series machine learning methods despite the temporal nature of both financial behavior and mental health conditions; sequential modeling approaches could enable early detection by capturing changes in financial patterns over time. Third, all ML studies were proof-of-concept demonstrations—none reported real-world deployment, discussed implementation barriers, or examined how predictions might facilitate clinical interventions.

More broadly, there appears to be scope for hybrid methodologies combining classical statistical tests for theory-testing with ML approaches for demonstrating practical applicability. The reviewed studies indicate a clear pattern in which statistical methods are primarily used for hypothesis testing, whereas machine learning approaches are more commonly applied for predictive objectives. Accordingly, researchers aiming to establish causal pathways or test hypotheses should employ statistical models with appropriate covariates, while those seeking to demonstrate practical utility—such as developing screening tools or early warning systems—may consider machine learning methods. Furthermore, the selection of machine learning evaluation metrics is critical, as high accuracy alone does not necessarily indicate clinical utility, particularly when output labels are imbalanced or definitions vary. Machine learning applications should also prioritize human-in-the-loop approaches that facilitate collaborative monitoring and decision-making by integrating model predictions with human judgment, clinical expertise, and contextual interpretation. Such approaches may improve transparency, trust, interpretability, and the practical utility of ML systems in mental health settings.

### 4.3 Limitations of self-report data

Open banking is facilitating an increase in digital finance (78, 79). This makes access to financial data possible for mental health research. Although there is some research on predicting psychological traits based on financial data (80–82), little is known about the potential of assessing mental health based on money behavior through objective financial data.

The overwhelming reliance on self-reported financial questionnaires in the studies in this review introduces several well-documented biases that compromise data quality. These include social desirability (underreporting financial difficulties due to stigma) (43), recall bias (inaccurate memory of past financial situations) (83), inherent selection bias (who chooses to participate in studies) (52, 76), and misclassification bias as a result of measurement error (69). Beyond measurement issues, questionnaire-based approaches face practical limitations, including time-intensive administration, limited sample sizes, and challenges related to translation and cultural adaptation across diverse languages (69, 84). These limitations constrain both the scale and precision of money-mental health research. However, there is also a need for mental health ground-truths even when using financial data sources for mental health assessment. Potential sources include real-world events (e.g., number of missed workdays due to mental illness), real world behavior (e.g., increased time at home, disrupted sleep), and healthcare events via Electronic Health Records (EHR).

### 4.4 The untapped potential of objective financial data

The heterogeneous nature of financial and mental health measurement instruments presents a further challenge, as differences in definitions, scales, and data collection methods limit comparability across studies and hinder development of generalizable models. This could be mitigated through objective financial data sources such as transaction records, combined with standardized subjective scales to provide behavioral context.

Only four studies leveraged objective data sources: an *N* = 1 pilot study analyzed bank transaction records to detect manic spending in bipolar disorder retrospectively (30), while three examined gambling platform data to identify problematic gambling patterns (39, 43, 44). This highlights an underexplored opportunity for future research. Objective financial data—such as transaction histories, spending patterns, and account activity—could potentially transform both research capabilities and clinical practice. This might not have been widely explored possibly because open banking recently became available.

Transaction-level data captures granular behavioral insights that questionnaires cannot, revealing subtle changes in financial behavior such as spending frequency, transaction timing, and purchase categories that could signal early symptomatic periods in conditions like bipolar disorder and ADHD where impulsivity manifests in observable spending patterns. This granularity could potentially enable early detection before crises occur, possibly supporting proactive rather than reactive intervention. Unlike heterogeneous questionnaire approaches, objective financial data provides standardized measurement across studies and populations. This also presents a complementary approach to digital phenotyping and current ecological momentary assessments (EMA), and can potentially improve their predictive power. Furthermore, transaction data can be collected passively via financial APIs (29), reducing participant burden compared to repeated questionnaire administration. In addition, objective financial data could help in providing interventions that assist in managing mental illness as well as the finances of people with mental illness.

Nevertheless, we note the multifaceted relationship between money and mental health, with (1) individual financial factors (e.g., financial status, history, difficulties) as an indicator of mental health, (2) financial behavior as a signal of symptomatic periods of mental illness, (3) economic factors influencing mental health (4) associated individual sociodemographic factors. Therefore, further research is needed to identify complementary data sources—such as socio and macroeconomic indicators—that may enhance insights beyond objective spending data alone.

### 4.5 The potential of money management interventions in mental illness

Money management interventions have been shown to improve the quality of life of people with mental illnesses (85). Digital mental health interventions such as just-in-time adaptive interventions (86), intelligent real-time therapy (87) and personalization strategies are often driven by analytical methods such as decision rules, data analytics and machine learning (88). While modeling techniques such as machine learning and statistical analysis are increasingly being explored in mental health (89, 90), the extent to which financial information is integrated into mental health assessment remains underexplored.

Machine learning has been recommended for delivering personalized digital mental health interventions (88). However, the use of machine learning in personalization interventions for digital mental health remains limited in practice, with systematic reviews indicating implementation rates of only 3–30% across interventions, and evidence predominantly derived from proof-of-concept prototypes and feasibility studies (88, 91). This highlights an important opportunity to further explore the integration of machine learning approaches and objective financial data within money management interventions.

Such approaches could support personalized budgeting, collaborative financial safeguards, and early identification of harmful financial behaviors, with the potential to improve both financial stability and mental health outcomes among individuals with mental illness. However, communicating mood states inferred from financial characteristics may also increase financial anxiety or distress. Human-centered research is therefore needed to determine how these interventions can be designed and delivered in ways that are acceptable, accessible, and psychologically safe.

Future research should also explore underexamined financial behaviors—including financial avoidance, hoarding, and prosocial spending—and their associations with mental health states. Finally, given the interwoven bidirectional relationship between finance and mental health, further research is needed to identify and evaluate policies that support financial management skills, financial literacy, and community-based social interventions.

### 4.6 Limited discussion of privacy and ethics

Most included studies did not substantively address privacy, data security, or ethical considerations beyond reporting institutional review board approvals. This likely reflects the field’s predominant reliance on self-report questionnaire data, which are often less sensitive and, in some cases, publicly available, compared with objective financial records. As the field moves toward using transaction-level data, comprehensive discussion of consent frameworks, data anonymization, algorithmic bias, and equitable access will become critical. Future primary research and reviews should explicitly examine these considerations. To realize the potential benefits of objective financial data in mental health research and to enable personalized financial management tools, further research is needed to identify and address the ethical, privacy, and security challenges associated with the use of such sensitive data. Computational ethics, an emerging field, may offer one promising avenue for continuous, automated oversight of digital phenotyping pipelines, moving beyond static institutional review toward continuous data governance. In addition, further research is also needed to strengthen the privacy and security of data and models, to build trust among individuals with mental illness and support practical clinical utility.

### 4.7 Strengths and limitations

Our scoping review examines the existing modeling techniques that have been explored in assessing financial variables and mental health. Other similar reviews (7, 31) have focused on reviewing the relationship, measurement approaches, and associations between debt, financial wellbeing, and mental health. To the best of our knowledge from extensive research in literature, this is the first review paper to systematically map analytical methods, and broad financial variables in the assessment of mental health.

#### 4.7.1 Search recency and scope

Nevertheless, our study has some limitations: our systematic search was completed in May 2024, meaning relevant papers published subsequently are not included. We have made substantial efforts to update the introduction (and literature review) to incorporate relevant publications that have appeared since the completion of data selection and extraction. Additionally, our keyword-based search strategy may have inadvertently excluded some relevant literature. For example, we did not include specialized economics databases such as EconLit, which may have resulted in the omission of a substantial body of economics and wellbeing research examining relationships between income, employment, debt, financial hardship, and subjective wellbeing or life satisfaction using large-scale surveys and longitudinal datasets. As a result, the review may underrepresent perspectives rooted primarily in economics, behavioral economics, and social policy literature (92). However, the selected databases were chosen to prioritize interdisciplinary coverage across mental health, health sciences, computing, and modeling research, in line with the primary objectives of the review, and are expected to have adequately captured literature within these domains. Similarly, studies from the banking and fintech sectors exploring transaction-based customer clustering (11) may not have appeared in our academic database searches due to publication in industry venues or use of different terminology. Future reviews could expand search strategies to include economic databases, gray literature, and industry publications.

#### 4.7.2 Exclusion of neurological conditions

We deliberately restricted our focus to mental health conditions as defined by standard diagnostic frameworks (DSM, ICD) and mental health themes like distress and wellbeing, excluding neurological disorders such as dementia where financial decision-making impairments are also documented (13). While this boundary ensured conceptual coherence, it may have excluded relevant methodological approaches applicable across cognitive and mental health conditions.

#### 4.7.3 Heterogeneity in outcomes and measures

The included studies examined diverse mental health conditions (e.g., depression, anxiety, bipolar disorder, ADHD, behavioral addictions), used varied assessment tools, and operationalized financial variables differently. While this heterogeneity reflects the breadth of the field, it precluded meta-analysis and limited our ability to make direct cross-study comparisons. Our synthesis therefore focuses on methodological patterns rather than pooled effect estimates.

#### 4.7.4 Potential for missed behavioral categories

Our search identified three primary clusters of financial factors: financial difficulty, specific behavioral patterns (gambling, impulsive spending) and macroeconomic factors. The relatively narrow range may reflect our keyword selection rather than the full scope of financial variables relevant to mental health.

Despite these limitations, our review provides a comprehensive foundation for understanding current methodological approaches and identifying critical gaps—particularly the underutilization of objective financial data, absence of temporal modeling, and limited exploration of machine learning methods—that represent key opportunities for advancing the field.

## 5 Conclusion

This scoping review provides the first comprehensive mapping of how money–mental health associations and analytical methods have been studied, revealing that the field remains largely reliant on self-reported financial measures despite the growing availability of objective transaction data. The reviewed literature supports a bidirectional relationship between mental health and financial factors, demonstrating consistent and significant associations between specific conditions—including depression, bipolar disorder, ADHD, and PTSD—and financial difficulties. Financial stressors exacerbate mental health conditions, while poor mental health, particularly in conditions like bipolar disorder and ADHD, often impairs financial management, perpetuating cycles of distress and economic instability. The review highlights that most studies have relied on statistical analyses of socioeconomic variables, with only one-fifth exploring machine learning approaches and a single study making use of objective financial data (e.g., bank statements), indicating a significant research gap.

These findings highlight a significant opportunity: financial behavioral data, particularly objective records such as bank transactions, could serve as digital biomarkers for mental health states. Advanced machine learning techniques applied to such data may enable passive, continuous assessment—moving beyond episodic self-report toward real-time monitoring. To enhance scalability and real-world applicability, future research should prioritize longitudinal studies with objective data, develop privacy-preserving modeling frameworks, and incorporate diverse socioeconomic and cultural contexts. By addressing these gaps, interdisciplinary approaches combining psychology, economics, and data science can inform targeted interventions and policies, fostering mental health resilience and financial empowerment across populations.
